# Development of a DDH Care Pathway for India: A Study Methodology to Guide Similar Efforts in Other Countries and for Other Conditions

**DOI:** 10.1007/s43465-021-00534-y

**Published:** 2021-10-22

**Authors:** Jacqueline Li, Alaric Aroojis, Kishore Mulpuri, Kevin G. Shea, Emily K. Schaeffer

**Affiliations:** 1 Department of Orthopaedics, University of British Columbia, Vancouver, BC Canada; 2 Department of Orthopaedic Surgery, BC Children’s Hospital, Vancouver, BC Canada; 3 Department of Paediatric Orthopaedics, Bai Jerbai Wadia Hospital for Children, Parel, Mumbai, Maharashtra 400012 India; 4 Department of Orthopaedic Surgery, Stanford University Medical Center, Palo Alto, CA USA

**Keywords:** Developmental dysplasia of the hip, Care pathway, Screening, Delphi process

## Abstract

**Purpose:**

In India and other Global South countries, developmental dysplasia of the hip (DDH) is often diagnosed after walking age, leading to more invasive surgeries and long-term disability. DDH care pathways aim to enhance early detection and must be tailored to meet a country’s needs and diverse practice settings. We describe a multi-phase methodology for context-specific DDH care pathway development, demonstrating its use in India.

**Methods:**

In Phase I, Orthopaedic surgeons, Pediatricians/Neonatologists, and Radiologists in India were surveyed regarding DDH screening. Seven relevant Indian organizations partnered together and assembled a multidisciplinary working group, which then met fortnightly to establish an evidence base and prepare for the subsequent consensus-building phase. During Phase II, panelists participated in a modified Delphi process to reach consensus on a list of DDH screening statements. Phase III applied the statements to develop the care pathway.

**Results:**

The Delphi process concluded after a preliminary survey and two Delphi rounds, reaching consensus on 47 statements, which were condensed into 35. The developed care pathway for India features periodic clinical hip examinations integrated with the country’s immunization schedule and selective imaging screening, providing flexibility in the timing and modality of imaging.

**Discussion/Conclusion:**

In Global South countries, there is a need for DDH care pathways specific to local contexts. Successful care pathway development requires accounting for cultural differences in healthcare and strategies to facilitate engagement and to address country-specific barriers. This methodology was feasible in India and can be applied to other conditions and/or countries wishing to establish care pathways.

**Level of Evidence:**

Level III.

## Introduction

Developmental dysplasia of the hip (DDH) represents a spectrum of hip abnormalities, present at birth or developed during infancy, that ranges from mild dysplasia to hip dislocation [1, 2]. This condition is a common cause of childhood disability and a leading cause of premature hip osteoarthritis requiring total hip replacement [3, 4]. Treatment options largely depend on the age of diagnosis and severity of the dysplasia [5, 6]. Hips diagnosed early (up to six months of age) can usually be managed non-operatively with abduction splinting [2, 6]. However, DDH with advancing severity or age necessitates more invasive surgical management [5, 6], increasing risk for poorer outcomes and complications, requiring further corrective surgeries.

Various screening programmes have been developed to improve the early detection of DDH [7]. While universal clinical examination is widely accepted, controversy exists surrounding the additional use of universal or selective ultrasound imaging for those with DDH risk factors [8–12]. Universal ultrasound screening may lead to unnecessary commitment of resources and over-treatment; however, long-standing universal ultrasound screening programmes in Austria and Germany have demonstrated success at reducing costs and the need for surgery [8, 11–14]. A recent systematic review, similarly, found support for universal ultrasound screening and its cost-effectiveness, though they cite important logistical and financial challenges that would hinder implementation of such a programme in India [15]. Despite ongoing debate over best screening practices, there is no doubt that all screening programmes reduce the late detection of DDH. Thus, especially in countries with limited resources, guidance that is offered must be practical within the region of interest and consider local practices and healthcare cultures to ensure feasibility.

A care pathway is a method used to guide or manage the care of a specific population of patients with a well-defined condition or problem for a well-defined period [16]. They are often developed by a multidisciplinary team, are evidence-based, and are meant to introduce and standardize patient-centered care [17]. Here, we define a DDH care pathway as a decision-making algorithm that guides healthcare providers through the process of screening—from clinical evaluation until referral to an orthopaedic surgeon or return to routine hip surveillance.

In India, the incidence of DDH is estimated to be between 1.0 and 9.2 per 1000 live births [18–20]. Recently, a survey of orthopaedic surgeons practicing in India found that over two-thirds of respondents had performed initial assessments on children with DDH older than one year of age [21]. Hence, a DDH care pathway for India, complemented by additional knowledge translation tools, would aid in the goal of standardizing patient care and enhancing early detection efforts across the country. The aim of this study was to develop a cost- and time-effective methodology to create a DDH Care Pathway for the Indian context, with the goal of detecting the majority of cases before walking age. We describe below the multi-phased approach, the use-case in India, and how it may be applied to guide efforts in other countries and for other conditions.

## Methodology and India Use-Case

### Overview of the Care Pathway Development Process

**Fig. 1:**
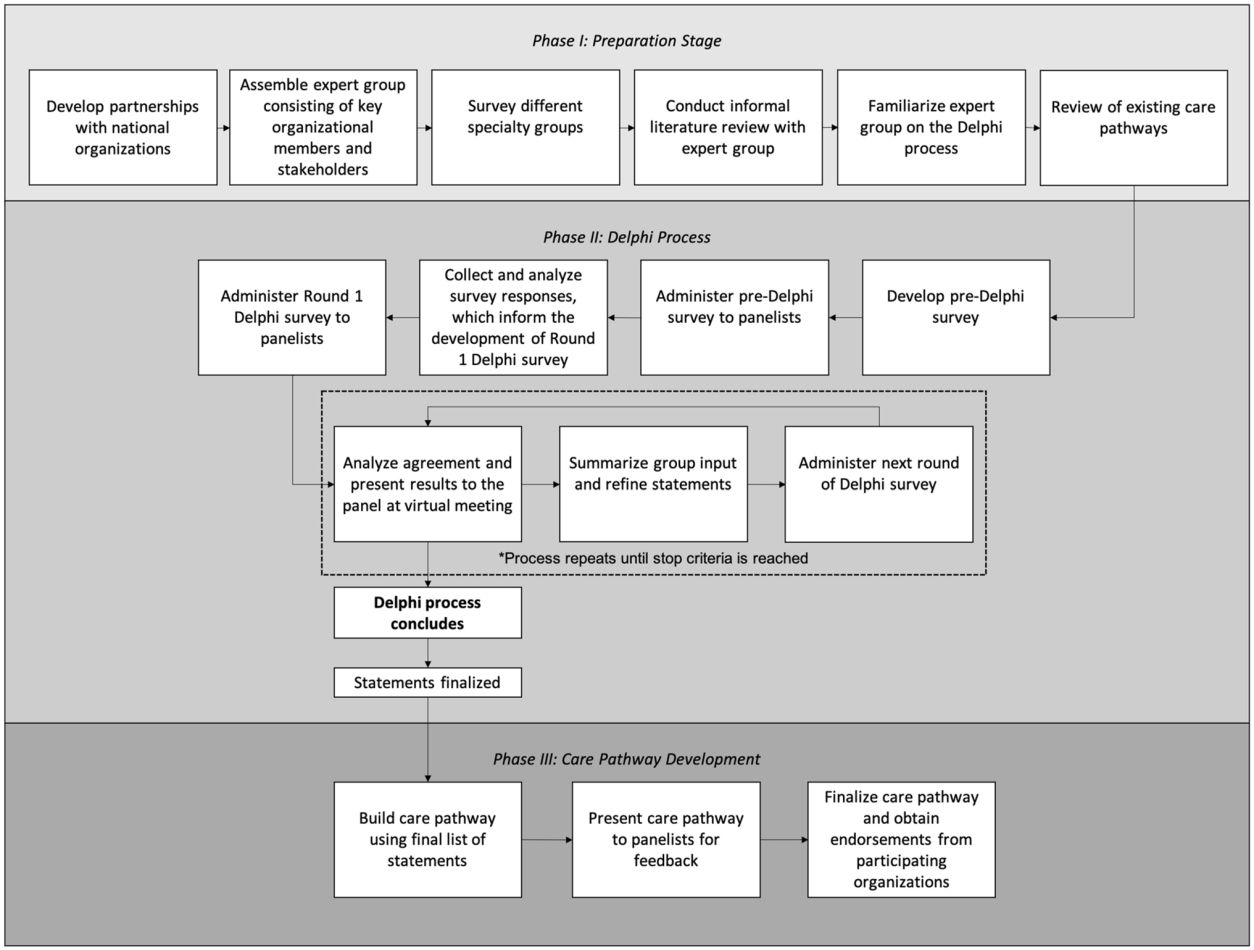
Flow diagram of the multi-phase process used for care pathway development. Adapted from Salkind NJ, eds. *Encyclopedia of Measurement and Statistics*. Thousand Oaks, CA: Sage Publications, Inc.; 2007: 242. https://doi.org/10.4135/9781412952644

The Pediatric Orthopaedic Society of India (POSI) and the Indian Academy of Pediatrics (IAP) led this joint initiative, with support from international collaborators. A three-phased approach was designed, requiring successful completion of the previous phase to continue into the subsequent phase (Fig. 1). Ethics approval was obtained by the coordinating institution for each phase of the project. Phase I focused on knowledge-building, increasing buy-in, and the enlistment of a working group in preparation for the following phase. In Phase II, panelists participated in a modified Delphi process to reach consensus on a comprehensive list of statements regarding DDH screening. In the final phase, a core writing group assembled the care pathway, which was reviewed by panelists and then endorsed by all participating organizations.

## Phase I

### Expert Group Formation

Key members from POSI and IAP were enlisted, and partnerships were then formed with the National Neonatology Forum of India (NNFI), Indian Radiological & Imaging Association (IRIA), Indian Federation of Ultrasound in Medicine & Biology (IFUMB), Federation of Obstetric & Gynaecological Societies of India (FOGSI), and Indian Orthopaedic Association (IOA)—all seven organizations representing over 120,000 healthcare providers in India. Each organization nominated representatives from various regions of the country to join the group based on their expertise on DDH and leadership within their respective organizations. The resulting expert group, designated as the DDH India Care Pathway Working Group, consisted of a group of orthopaedic surgeons, pediatricians, neonatologists, gynaecologists, and radiologists, reflecting the range of physician stakeholders relevant to the care of DDH patients in India. In view of travel restrictions imposed by the COVID-19 pandemic in 2020–2021 and the geographical distribution of the group members, meetings were conducted virtually using the Zoom platform (Zoom Video Communications, Inc. 2012, Version 5.0, San Jose, CA, USA). Email and web applications were used to facilitate communication between group members.

### Initial Survey of Specialty Groups

Upon forming partnerships, the members of POSI, IAP, NNFI, IRIA, and IFUMB were surveyed to understand the scope of options available to providers in India prior to consensus-building. Surveys were developed in collaboration with international investigators and local experts and asked about DDH resource availability, current screening and referral pathways, attitudes towards care pathways, and other discipline-specific questions. Surveys were administered using the Research Electronic Data Capture (REDCap) tool, a secure web application designed for building and managing online surveys and databases hosted at the coordinating institution (REDCap. 2004, Version 10.6.25, Vanderbilt University, Nashville, TN, USA) [22, 23]. Preliminary survey findings demonstrated good access to ultrasound and X-ray; however, they suggested potential variability in the quality and reliability of ultrasound reporting, complicating the existing debate on selective versus universal ultrasound screening and necessitating further discussions on care pathway flexibility to account for this variability. The surveys also demonstrated low awareness about DDH among pediatricians/neonatologists, and limited experience with clinical and imaging tools for diagnosis. To improve awareness, infographics outlining the incidence of DDH, risk factors, diagnostic tools, and the importance of early detection and management were developed and circulated to organizational members.

### Literature Review

Group members participated in a three-month literature review process through a series of fortnightly meetings, which included scheduled presentations by group members followed by questions and discussion. Presenters synthesized relevant high-quality articles on designated topics, including the incidence of DDH in India, clinical examination, the role of imaging, and comparisons of existing screening programmes. An online repository of comprehensive literature was also created for the group and stored on a cloud-based server for ready reference.

### Preparatory Meeting for Consensus-Building

Upon completion of the literature review, the consensus-building phase was introduced. The external facilitator reviewed existing care pathways and explained the upcoming modified Delphi approach, outlining past uses of the Delphi along with the panelist’s role, significance, and extent of their expected commitment during Phase II.

## Phase II

### Overview of the Delphi Process

The aim of Phase II was to reach consensus on a comprehensive list of statements for the screening of DDH from birth until walking age in India. To achieve this, the Delphi approach, which is a consensus-building method requiring iterative surveys and controlled feedback, was used [24]. The modified Delphi process used in this study employed online rounds of surveys, along with virtual meetings between each round, until a pre-designated stopping point was reached (Fig. 1).

Each Delphi survey consisted of a list of consensus statements regarding key components of DDH screening. Statements were rated on a five-point Likert scale and 80% was set as the threshold of consensus for a given statement (≥ 80% of respondents selecting “agree” or “strongly agree”). Respondents could also select “N/A” (excluding them from the denominator) if they felt that they lacked the expertise to provide a rating. Each consensus statement was followed by an optional comment box for respondents to address concerns, propose changes, ask questions, and/or explain the rationale for their selection.

All surveys were administered using REDCap and distributed by email and WhatsApp messaging platform. Panelists were given 2 weeks to complete each survey—non-respondents were sent reminders throughout the period. Those who were unable to complete the survey in the designated time frame were excluded from the round and any following rounds; however, their survey results from previous round(s) were retained for analysis.

At each virtual meeting, the external facilitator presented the aggregate results and the panelists discussed the statements, focusing on ones that did not reach consensus. Panelists unable to attend the meeting could view a recorded version of the meeting or a slide deck containing the results and summary of the discussion. After each round, statements that reached consensus were incorporated into the final list. Statements were also modified, added, removed, or brought back in the subsequent round based on group input and the discretion of the investigators. A priori, it was determined that the Delphi process would conclude when a) ≥ 90% of cumulative items reached consensus or b) after four iterations of the Delphi survey were completed.

### Preliminary “pre-Delphi” Survey

**Fig. 2:**
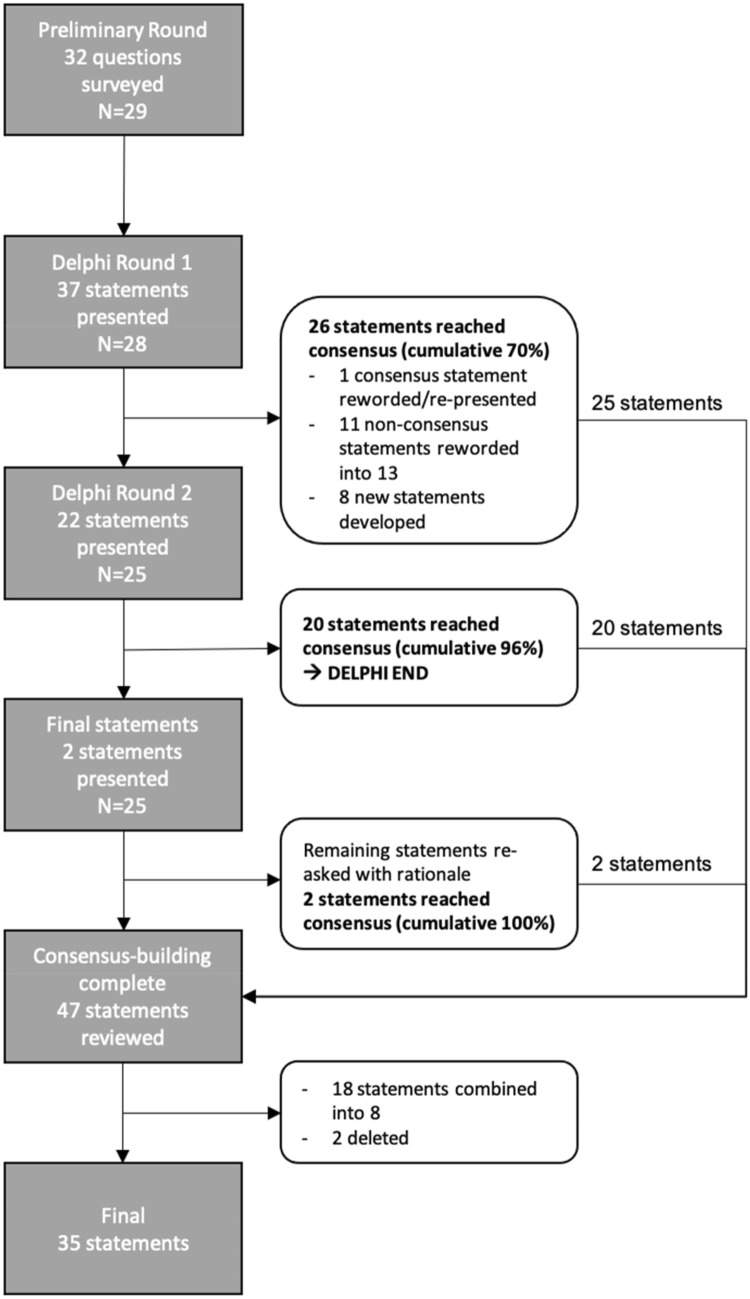
Flow diagram of the consensus-building process for DDH care pathway development in India. N = number of survey respondents

The preliminary survey consisted of 32 questions and served as the foundation for the subsequent Delphi survey (Fig. 2). Panelists were asked about their practice characteristics and opinions regarding DDH screening: risk factors, clinical exam, ultrasound, X-ray, and timing of referral. The demographic characteristics of the 29 respondents are summarized in Table 1.

**Table 1:** Demographic characteristics of the respondents to the preliminary Delphi survey

	N (%)
Clinical occupation	
Orthopaedic surgeon	10 (34.5%)
Pediatrician	10 (34.5%)
Radiologist	4 (13.8%)
Gynaecologist	3 (10.3%)
Neonatologist	2 (6.9%)
Organization representation	
Pediatric Orthopaedic Society of India	9 (31.0%)
Indian Academy of Pediatrics	9 (31.0%)
Indian Radiological and Imaging Association	2 (6.9%)
National Neonatology Forum of India	3 (10.3%)
Indian Federation of Ultrasound in Medicine and Biology	2 (6.9%)
Federation of Obstetric and Gynaecological Societies of India	3 (10.3%)
Indian Orthopaedic Association	1 (3.4%)
Private vs public sector experience	
Private	9 (31.0%)
Public	5 (17.2%)
Both	15 (51.7%)
Solo vs group setting	
Solo	11 (37.9%)
Group	16 (55.2%)
Partnership	1 (3.4%)
Other	1 (3.4%)
Urban vs rural setting	
Urban	28 (96.6%)
Urban and rural	1 (3.4%)

The preliminary survey favored universal clinical screening (69%) with age-dependant clinical tests and selective imaging using either ultrasound (97%) or X-ray (93%). Similar to the specialty group surveys, ultrasound and X-ray were available; however, there was variability in opinion on the age range for ultrasounds and lower age limit for X-rays. However, all respondents agreed that the availability of quality ultrasound reporting and interpretation must be considered when determining imaging modality. The survey also highlighted the need to incorporate trained paramedical personnel as potential users of the care pathway, including auxiliary nurse midwives, accredited social health workers, mobile health teams at the community level, and medical teams at district early intervention centers and district hospitals.

### Delphi Round 1

The results of the preliminary survey were used to frame consensus statements for Round 1, which consisted of 37 statements. In total, 28/29 (97%) preliminary respondents completed the survey, with 70% (26/37) of statements reaching consensus, thus necessitating another Delphi round. Following the group meeting, eight new statements were developed, 11 statements that had not been agreed upon were re-worded into 13 statements, and one statement which was previously agreed upon was also re-worded and included (Fig. 2).

### Delphi Round 2

Round 2 consisted of 22 statements and was completed by 25/28 (89%) respondents from Round 1. An additional 20 statements reached consensus, for a cumulative 96% (45/47), thus meeting the criteria to conclude the Delphi process (Fig. 2). Another meeting was held to share the results, discuss the two remaining statements that did not reach consensus, and then announce the conclusion of the Delphi process.

### Statement Finalization

The two remaining statements were re-distributed along with a rationale/summary of the group discussion. All 25 participants who completed Round 2 responded, with both statements reaching consensus. These statements were then incorporated into the list, for a total of 47 consensus statements (Fig. 2).

Three experts (JL, EKS, AA) then reviewed the complete list of statements and divided them into five categories. During this process, 18 statements were simplified into eight statements and two statements were deleted, resulting in a final list of 35 statements (Table 2).

**Table 2:**
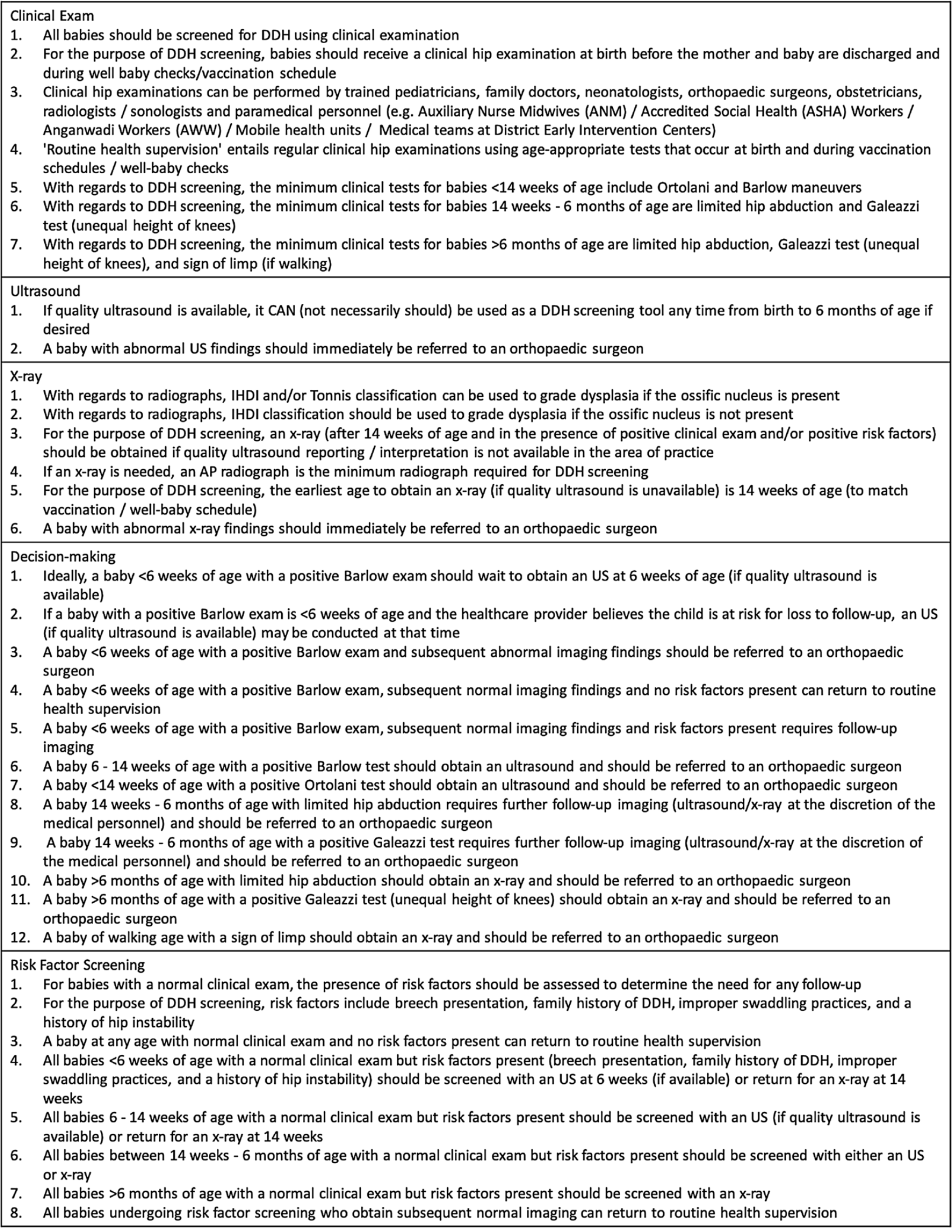
Final list of 35 consensus statements developed for the DDH India Care Pathway subdivided into five categories

## Phase III

In the third phase, a core writing group applied the consensus statements to create the DDH care pathway algorithm for India and a written version. These materials were circulated to the expert panel for revisions. A final meeting was held to review the care pathway before finalization and formal endorsement from all organizations (Fig. 1).

The conclusion of the consensus-building process resulted in a comprehensive algorithm for the screening of DDH in India, from birth until walking age. The tool reflects the current expert-based consensus among physicians in India and has been designed as a primary guide for use by local healthcare providers. Key features of the care pathway are shown in Table 3.

**Table 3:**
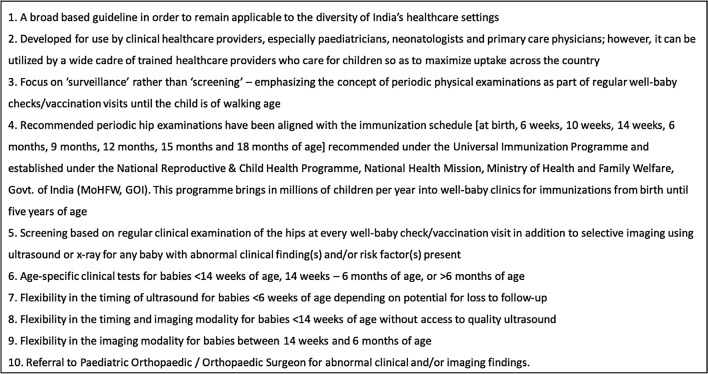
Features of the DDH Care Pathway for India

## Discussion

This study demonstrates the successful development and application of a feasible methodology for the creation of a DDH care pathway for India. With a growing population of over 1.3 billion people and the goal of nationwide uptake, the development of a novel care pathway was essential, as established programmes and care pathways in the Global North lack utility in the complex Indian healthcare setting. The care pathway is applicable in the public and private healthcare sectors, and the nation’s diverse practice settings and geographic locations, including rural areas which account for approximately 69% of India’s population [25]. To our knowledge, no other study has used a Delphi process to develop a care pathway for DDH screening. However, a DDH care pathway has been developed for the St. Luke’s Health System in Idaho, USA, considering the state’s geographic characteristics and rural population [26]. The pathway provides primary care practitioners and pediatricians with a simple algorithm to manage each case. While the pathway is designed to standardize patient care and enhance the early detection of DDH throughout the health system, it also allows for flexibility, providing alternative pathways for those lacking access to ideal imaging, and ensuring that all children in the health system receive quality DDH screening regardless of their location or life circumstance [26].

In 2019, Kelley et al. used the Delphi method to reach consensus on a set of principles for the management of DDH with a Pavlik harness [27]. Similarly, Roposch et al. (2011) established international consensus on diagnostic criteria for DDH in early infancy using a Delphi process; however, the consensus gained from these studies inform practice and emphasize global as opposed to regional or country-specific guidance [27, 28].

The Delphi method was ideal for this study because of the existing controversy surrounding best practices for DDH screening, necessitating a methodology that integrates evidence in the literature with the best judgment of experts. Additionally, the ability to conduct the process virtually resolved the logistical and financial challenges of facilitating meetings with a large and geographically diverse panel. Our involvement of experts from diverse settings and specialties during consensus-building has been recommended to bring in alternative viewpoints and ensure a wide knowledge base [29, 30]. Obtaining support from leaders and stakeholders from multiple organizations will also help to increase future buy-in and uptake of the care pathway in India. It was also essential that the composition of the expert panel accounted for cultural differences in healthcare. The target users are primarily pediatricians and neonatologists and hence, involving IAP and NNFI alongside POSI was crucial to the success of this project. Considering the variability in the quality and reliability of ultrasound reporting and restrictive access to ultrasound due to the Pre-Conception and Pre-Natal Diagnostic Techniques (PCPNDT) Act, 1994 banning pre-natal sex determination in India, it was also important to involve sonologists and radiologists to provide feedback on the ideal imaging modality for DDH screening. Additionally, although obstetrician-gynaecologists may not be relevant to the care of DDH patients in many Global North countries, their participation was important in the Indian healthcare setting as a frequent first point of contact with newborns and their families. In addition to serving as potential examiners of newborn hips, obstetrician-gynaecologists are an important resource for families in India, often guiding parents on various aspects of newborn care, such as hip-safe swaddling practices.

Multiple measures were put in place to manage the inherent challenges resulting from group diversity, such as the ability to opt out of responding to Delphi statements. Additionally, the literature review allowed all group members to become sensitized to the literature. The foundational knowledge base developed prior to solicitation of group members’ opinions may have contributed to the generally high levels of agreement in the preliminary survey and resulted in fewer Delphi rounds; however, the extent of review included in the methodology is unique to the group and may not always be necessary.

The consideration of facilitators and barriers to development is key to successful care pathway implementation [31]. Phase I allowed the group to establish rapport and ensured preparedness for the Delphi process. The surveys during this phase also identified critical knowledge gaps that will be addressed during implementation, including deficits in pediatricians’ and neonatologists’ knowledge about hip-safe swaddling, risk factor screening, and various clinical tests. It also became apparent that bringing children in solely for hip examinations would be challenging, resulting in the alignment of the recommended hip examination schedule with the country’s immunization schedule (Table 3). An important consideration for future care pathways will be to leverage existing infrastructure or programs to simplify implementation and maximize uptake. Additionally, while group members in this study were highly motivated, investigators must be wary of declining motivation and implement appropriate strategies to facilitate and maintain engagement.

One limitation of the use-case in India was the lack of representation from nursing associations, parent groups, and physicians practicing in rural areas in the working group. Although the Indian population continues to urbanize, measures must be taken to ensure that babies in rural areas are not overlooked, as their risk for late detection may be greater due to limited access to health resources. However, the Delphi statements and resulting algorithm did address variability in resource accessibility at every step of the care pathway. This included permitting a wide variety of trained physicians and paramedical personnel to perform clinical examinations and providing flexibility in the timing and/or modality of imaging (Table 3). Nurses and physicians with rural experience will also be involved during implementation and future care pathway revisions. Another inherent limitation of the methodology was the lack of true anonymity during consensus-building [32]. This was mitigated by collecting survey responses outside of the meeting time and presenting aggregate results. Additionally, the success of the initiative relies on the presence of a local champion in the country to serve as the Project Lead. This individual must have the experience, expertise, passion, and positional leadership to unify all relevant organizations and motivate group members through all phases to ensure that the project goals are met.

There is an urgent need for action to reduce global inequities in DDH screening. A context-specific DDH care pathway such as this has the potential to benefit millions of infants born each year in the country and globally. The cost- and time-effective methodology we have described can be used to develop care pathways in other countries and may be particularly useful in regions with limited funds and other resources. Recently, this approach was successfully used to develop a hip surveillance guideline for children with cerebral palsy in India—this methodology can continue to be applied to other conditions with a similarly large burden and controversy regarding best practices.

## Conclusion

The late detection of DDH in Global South countries such as India necessitates global health initiatives to combat this. The three-phased methodology outlined in this paper requires a preparatory phase, a consensus-building phase using a modified Delphi approach, and a care pathway development/writing phase. The methodology was successfully used to develop a DDH care pathway in India, demonstrating feasibility given the limited funds available and the nation’s diverse health landscape. Other countries can apply this methodology to develop DDH care pathways, or care pathways for other conditions, that are unique to their local context.
